# Educational Health Disparities in Cardiovascular Disease Risk Factors: Findings from Jamaica Health and Lifestyle Survey 2007–2008

**DOI:** 10.3389/fcvm.2017.00028

**Published:** 2017-05-15

**Authors:** Trevor S. Ferguson, Novie O. M. Younger-Coleman, Marshall K. Tulloch-Reid, Ian R. Hambleton, Damian K. Francis, Nadia R. Bennett, Shelly R. McFarlane, Aurelian Bidulescu, Marlene Y. MacLeish, Anselm J. M. Hennis, Rainford J. Wilks, E. Nigel Harris, Louis W. Sullivan

**Affiliations:** ^1^Epidemiology Research Unit, Caribbean Institute for Health Research, The University of the West Indies, Kingston, Jamaica; ^2^Chronic Disease Research Centre, Caribbean Institute for Health Research, The University of the West Indies, Bridgetown, Barbados; ^3^Indiana University School of Public Health – Bloomington, Bloomington, IN, USA; ^4^Department of Medical Education, Morehouse School of Medicine, Atlanta, GA, USA; ^5^Department of Non-Communicable Diseases and Mental Health, Pan American Health Organization, Washington, DC, USA; ^6^The University of the West Indies, Kingston, Jamaica; ^7^The Sullivan Alliance, Alexandria, VA, USA

**Keywords:** health disparity, cardiovascular disease, education, socioeconomic status, hypertension, diabetes, obesity, hypercholesterolemia

## Abstract

**Objectives:**

Socioeconomic disparities in health have emerged as an important area in public health, but studies from Afro-Caribbean populations are uncommon. In this study, we report on educational health disparities in cardiovascular disease (CVD) risk factors (hypertension, diabetes mellitus, hypercholesterolemia, and obesity), among Jamaican adults.

**Methods:**

We analyzed data from the Jamaica Health and Lifestyle Survey 2007–2008. Trained research staff administered questionnaires and obtained measurements of blood pressure, anthropometrics, glucose and cholesterol. CVD risk factors were defined by internationally accepted cut-points. Educational level was classified as primary or lower, junior secondary, full secondary, and post-secondary. Educational disparities were assessed using age-adjusted or age-specific prevalence ratios and prevalence differences obtained from Poisson regression models. Post-secondary education was used as the reference category for all comparisons. Analyses were weighted for complex survey design to yield nationally representative estimates.

**Results:**

The sample included 678 men and 1,553 women with mean age of 39.4 years. The effect of education on CVD risk factors differed between men and women and by age group among women. Age-adjusted prevalence of diabetes mellitus was higher among men with less education, with prevalence differences ranging from 6.9 to 7.4 percentage points (*p* < 0.05 for each group). Prevalence ratios for diabetes among men ranged from 3.3 to 3.5 but were not statistically significant. Age-specific prevalence of hypertension was generally higher among the less educated women, with statistically significant prevalence differences ranging from 6.0 to 45.6 percentage points and prevalence ratios ranging from 2.5 to 4.3. Similarly, estimates for obesity and hypercholesterolemia suggested that prevalence was higher among the less educated younger women (25–39 years) and among more educated older women (40–59 and 60–74 years). There were no statistically significant associations for diabetes among women, or for hypertension, high cholesterol, or obesity among men.

**Conclusion:**

Educational health disparities were demonstrated for diabetes mellitus among men, and for obesity, hypertension, and hypercholesterolemia among women in Jamaica. Prevalence of diabetes was higher among less educated men, while among younger women the prevalence of hypertension, hypercholesterolemia, and obesity was higher among those with less education.

## Introduction

Health disparities have become an important area of focus in public health research, practice, and policy development (1). Studies in the United States and the United Kingdom demonstrate significant racial/ethnic health disparities, with poorer health for black populations when compared to white populations (2, 3). Socioeconomic health disparities are another area of concern, due to reports showing poorer health among persons in lower occupation categories and among persons with lower educational attainment (4, 5). In the United States, the elimination of health disparities was a major goal of the Healthy People 2010 initiative, and this has been extended to Healthy People 2020 (6–8). Globally, The World Health Organization (WHO) has led efforts to highlight social determinants of health, through a special report in 2005 (9) and an international conference in 2011 which culminated with a political declaration calling for action on the social determinants of health (10, 11). With the growing understanding that social factors are important drivers of health inequality, more studies examining the effects of these factors on disease burden in low- and middle-income countries are needed (12–14). In addition to providing critical data for developing health and social policy, these data can improve our understanding of the mechanisms underlying these socioeconomic health disparities.

Cardiovascular disease (CVD) is the leading cause of death globally accounting for over 17 million or 31% of global deaths (15). In Jamaica and other Caribbean countries, CVD account for four of the five leading causes of death (16). Additionally, there is a high prevalence of CVD risk factors in Jamaica, with 52% of the population being overweight or obese, 25% having hypertension, 12% having hypercholesterolemia, and 8% having diabetes (17).

Several studies have shown that the burden of CVD and its risk factors vary with socioeconomic status (4). While developed countries report an inverse relationship between socioeconomic status and CVD (4, 18), the relationship is less consistent in low- and middle-income countries, with some studies showing higher risk among persons of higher SES (19–21). Previous studies from Jamaica suggest that the impact of SES on CVD risk differs in men and women (22–26). Most of the data for the studies from Jamaica were collected about 20 years ago, and none of these studies used nationally representative samples. We therefore aimed to evaluate the extent of socioeconomic health disparity in CVD risk factors [high blood pressure (BP), diabetes, hypercholesterolemia, and obesity] among a nationally representative sample of Jamaican adults using education as the measure of socioeconomic status.

## Materials and Methods

### Study Sample and Procedures

The study was conducted using data from the second Jamaica Health and Lifestyle Survey conducted in 2007–2008. Details of the methods used in these surveys have been previously published (27–29). The survey enrolled a nationally representative sample of 2,848 Jamaicans, aged 15–74 years, with a response rate of almost 98% (28).

Participants were selected using a multi-stage sampling method. The primary sampling units (PSUs) were enumeration districts selected using probability proportionate to size. A random household was selected as the starting point within each PSU; thereafter, other households were selected systematically, at intervals determined by the number of households in the PSU, in order to obtain a similar number of households per PSU. Within each household, one participant was selected using the Kish random selection method (30). If the selected household member declined to participate, this was counted as a non-response and the next household was visited to recruit a participant.

The study protocol was reviewed and approved by the Ethics Committees of the Faculty of Medical Sciences of the University of the West Indies, Mona, and the Ministry of Health, Jamaica. All participants provided written informed consent.

### Variables: Measurement and Definitions

Data were collected by trained observers in face-to-face interviews. The outcome variables assessed were hypertension, diabetes mellitus, hypercholesterolemia, and obesity. The health disparity variable was educational attainment, while age and sex were treated as confounders or effect modifiers. Education was chosen as the measure of socioeconomic status as data on education were available for almost all study participants, and previous studies have shown significant associations between educational attainment and CVD risk factors (4, 24–26). Data on income were unavailable for approximately 30% of participants, while data on occupation were unavailable for approximately 10% of participants.

#### Assessment of CVD Risk Factors

Blood pressure was measured using a mercury sphygmomanometer and followed a standardized protocol (31). Three BP measurements were taken at 1-min intervals using the right arm after the participant had been seated for 5 min. The mean of the second and third BP measurements was used in the analysis. Hypertension was defined using criteria from the Seventh Report of the Joint National Committee on Prevention, Detection, Evaluation and Treatment of High Blood Pressure (JNC 7) as BP ≥ 140/90 mmHg or being on medication for high BP (32).

Body weight was measured to the nearest 0.1 kg using a portable digital scale, while height was measured to the nearest 0.1 cm using a portable stadiometer. Instruments were calibrated weekly. Body mass index (BMI) was calculated as weight in kilograms divided by the square of height in meters and categorized using the WHO BMI categories, with obesity defined as BMI ≥ 30.0 kg/m^2^ (33).

Fasting blood glucose and total cholesterol were measured after a 10-h overnight fast using capillary blood samples and analyzed with a portable point-of-care device (Accutrend^®^ GCT Roche Diagnostics GmbH). For diabetes, measured capillary glucose was converted to the equivalent fasting plasma glucose using the formula “plasma glucose = 0.102 + 1.066 × capillary glucose” as recommended by the guidelines from the European Association for the Study of Diabetes (34). Diabetes was defined as fasting glucose ≥7.0 mmol/L or being on medication for diabetes in accordance to the WHO/American Diabetes Association criteria (35, 36). High cholesterol was defined as fasting total cholesterol of ≥5.2 mmol/L (37).

#### Categorization of Educational Attainment

Data on education were collected by self-report as the highest level of education completed and divided into four categories, namely, “primary or lower” (up to grade 6), “junior secondary” (up to grade 9), full secondary (at least grade 11), and “post-secondary” (vocational training, college, or university). Analyses were limited to participants 25 years and older, since the younger participants may not have completed secondary or tertiary level education.

### Statistical Analyses

Statistical analyses were performed using Stata 12.1 statistical software (Stata Corp., College Station, TX, USA). Reported estimates were weighted to account for multi-stage survey design. Descriptive analyses yielded means or proportions for demographic variables, CVD risk factors, and education level. We obtained crude- and sex-specific estimates of the prevalence for the CVD risk factors and then category-specific estimates within education and age categories. Age-adjusted prevalence estimates, prevalence difference, and prevalence ratios were obtained using Poisson regression models. Prevalence ratios were used in this study because it has been shown to be a more accurate measure of effect, compared to the odds ratio, in studies where the prevalence of the outcome is high (38, 39). Separate models were created for each outcome and post-estimation commands were used to derive adjusted estimates. Sex and age group interactions were tested in the regression models and included in the final models if statistically significant. There was evidence for sex interaction in the relationship between CVD risk factors and education; additionally, there was age interaction in the relationship between education and CVD risk factors among females, but not among males. We therefore presented sex-specific age-adjusted models for males and age-specific models for females. Age-specific estimates were derived from models which included the interaction terms. In addition to age adjustment, final models were adjusted for other covariates based on the level of significance; variables with *p* values <0.2 were kept in the final model (40). Covariates included in each model are shown in the table footnotes. Analyses were limited to participants with complete data on education, hypertension, and diabetes mellitus.

## Results

The analyzed sample included 2,231 participants (678 men and 1,553 women) with mean age of 39.4 years. The characteristics of the survey participants are shown in Tables 1 and 2. Men were marginally older than women and had higher mean height and BP. Women had higher mean BMI, fasting glucose, and cholesterol. The overall prevalence of hypertension was 26%, while the prevalence of diabetes was 8.0%. Women had statistically significant higher prevalence of hypercholesterolemia (26 vs. 8%, *p* < 0.001) and obesity (43 vs 12%, *p* < 0.001). The largest education category was for those with full secondary level education, 46% overall; 16% had post-secondary education and 12% had completed only primary level education. There was a statistically significant sex difference in the distribution of education (*p* < 0.001) with a higher proportion of women having completed secondary or post-secondary education.

**Table 1:** **Mean values of characteristics for 25- to 74-year-old Jamaicans by sex (Jamaica Health and Lifestyle Survey 2007–2008)**.

Characteristic	Men *n* = 678 Mean (SE)	Women *n* = 1,553 Mean (SE)	Total *N* = 2,231 Mean (SE)
Age (years)***	40.3 (0.12)	38.7 (0.07)	39.4 (0.07)
Height (m)***	1.76 (0.003)	1.61 (0.003)	1.68 (0.002)
Weight (kg)	76.1 (0.94)	77.2 (0.72)	76.7 (0.61)
Body mass index (kg/m^2^)***	24.7 (0.28)	29.6 (0.34)	27.4 (0.24)
Systolic blood pressure (mmHg)***	128.7 (0.57)	123.1 (0.61)	125.6 (0.47)
DBP (mmHg)***	80.5 (0.49)	77.7 (0.71)	79.0 (0.49)
Glucose (mmol/L)***	4.23 (0.10)	4.64 (0.05)	4.45 (0.06)
Cholesterol (mmol/L)***	4.26 (0.02)	4.63 (0.02)	4.46 (0.01)

***p < 0.001 for male:female difference.

*Estimated means are weighted for complex survey design to provide nationally representative (population) estimates; therefore, we report SEs instead of SD*.

*N for cholesterol estimates = 2,087 and for glucose estimation = 2,113*.

**Table 2:** **Proportion of Jamaicans 25–74 years old in education categories and with individual cardiovascular disease risk factors (Jamaica Health and Lifestyle Survey 2007–2008)**.

Characteristic	Men *N* = 678 % (*n*)	Women *N* = 1,553 % (*n*)	Total *N* = 2,231 % (*n*)
Education categories***			
Post-secondary	10.4 (64)	21.1 (136)	16.3 (200)
Full secondary	38.7 (268)	51.9 (734)	46.0 (1,002)
Junior secondary	37.8 (200)	16.8 (411)	26.2 (611)
Primary or lower	13.1 (146)	10.2 (272)	11.5 (418)
Hypertension	26.8 (250)	24.5 (616)	25.6 (866)
Diabetes	6.7 (71)	8.8 (221)	7.9 (292)
High cholesterol***	8.3 (67)	26.1 (311)	18.1 (378)
Obesity***	11.5 (82)	42.6 (699)	28.6 (781)

***p < 0.001 for male:female difference.

*n = number of participants providing data for category. Proportions are weighted for complex survey design to provide nationally representative (population) estimates. Percentages shown reflect the weighted estimates and not the simple proportion of participants based on observed numbers*.

*N for cholesterol estimates = 2,090 (males 636, females 1,454)*.

In order to assess the potential effect of participants excluded due to missing data (96/2,327), we compared prevalence of CVD risk factors and educational attainment for included vs. excluded participants. Except for a lower prevalence of hypertension among excluded participants (14 vs. 26%, *p* = 0.012), there were no statistically significant differences in the prevalence or CVD risk factors or educational attainment for those excluded from analyses compared to those included in the analyses.

**Figure 1:**
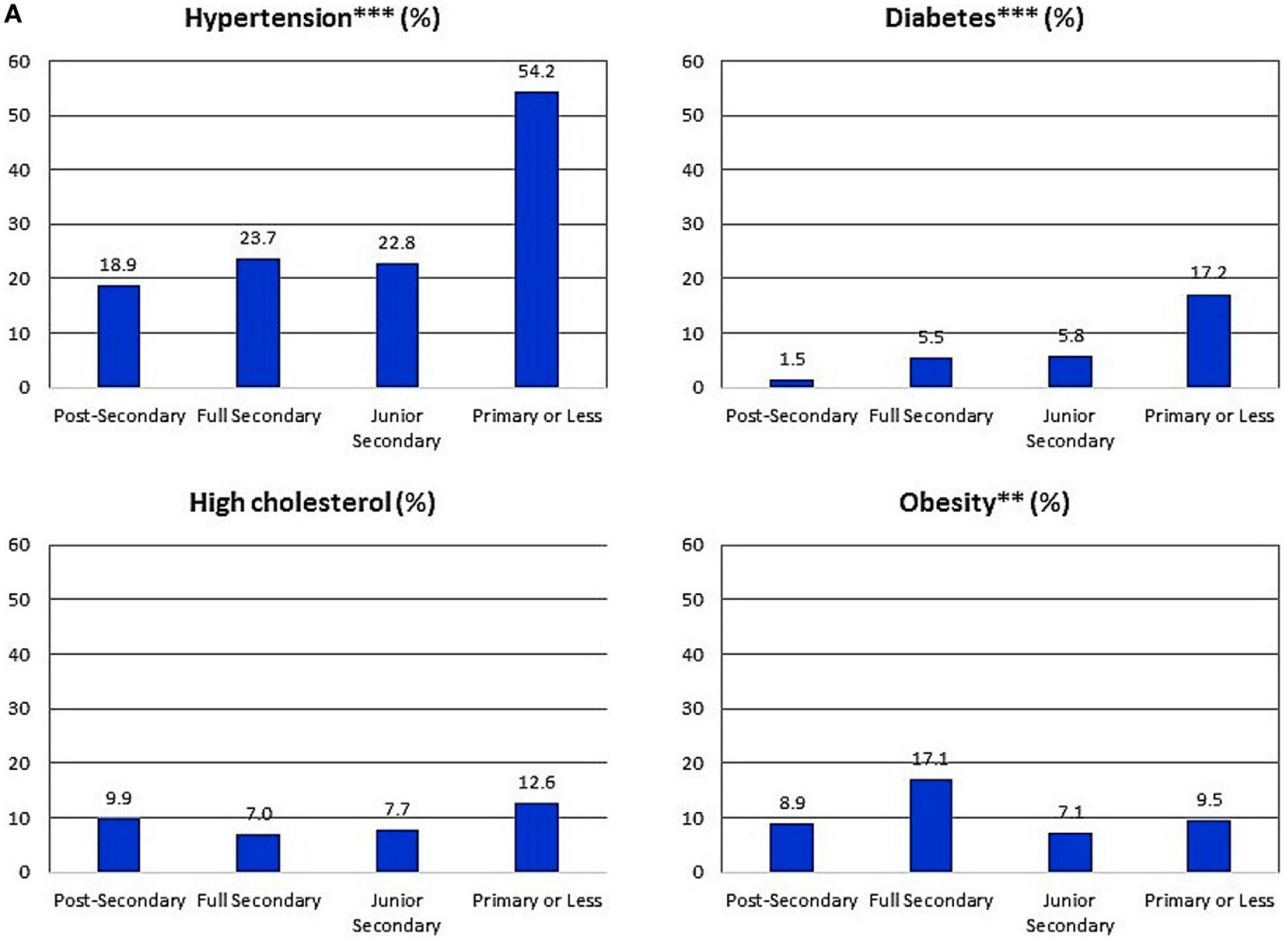

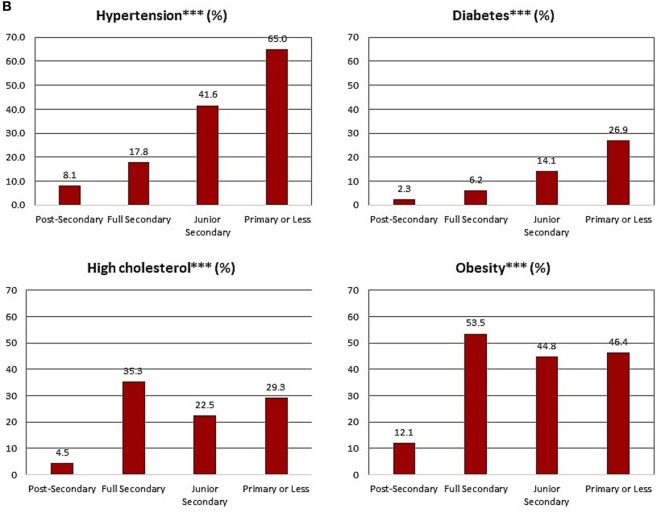
**(A)** Proportion of Jamaicans men 25–74 years old with individual cardiovascular disease (CVD) risk factors various within education categories (Jamaica Health and Lifestyle Survey 2007–2008). **p < 0.01; ***p < 0.001 for difference in proportion across education categories, derived from chi-squared tests. Proportions are weighted for complex survey design to provide nationally representative (population) estimates. Percentages shown reflect the weighted estimates and not the simple proportion of participants based on observed numbers. *n* = 64 for post-secondary education, 268 for full secondary education, 200 for junior secondary education, and 146 for primary or less education. **(B)** Proportion of Jamaicans women 25–74 years old with individual CVD risk factors various within education categories (Jamaica Health and Lifestyle Survey 2007–2008). **p < 0.01; ***p < 0.001 for difference in proportion across education categories, derived from chi-squared tests. *n* = 136 for post-secondary education, 734 for full secondary education, 411 for junior secondary education, and 272 for primary or less education.

**Figure 2:**
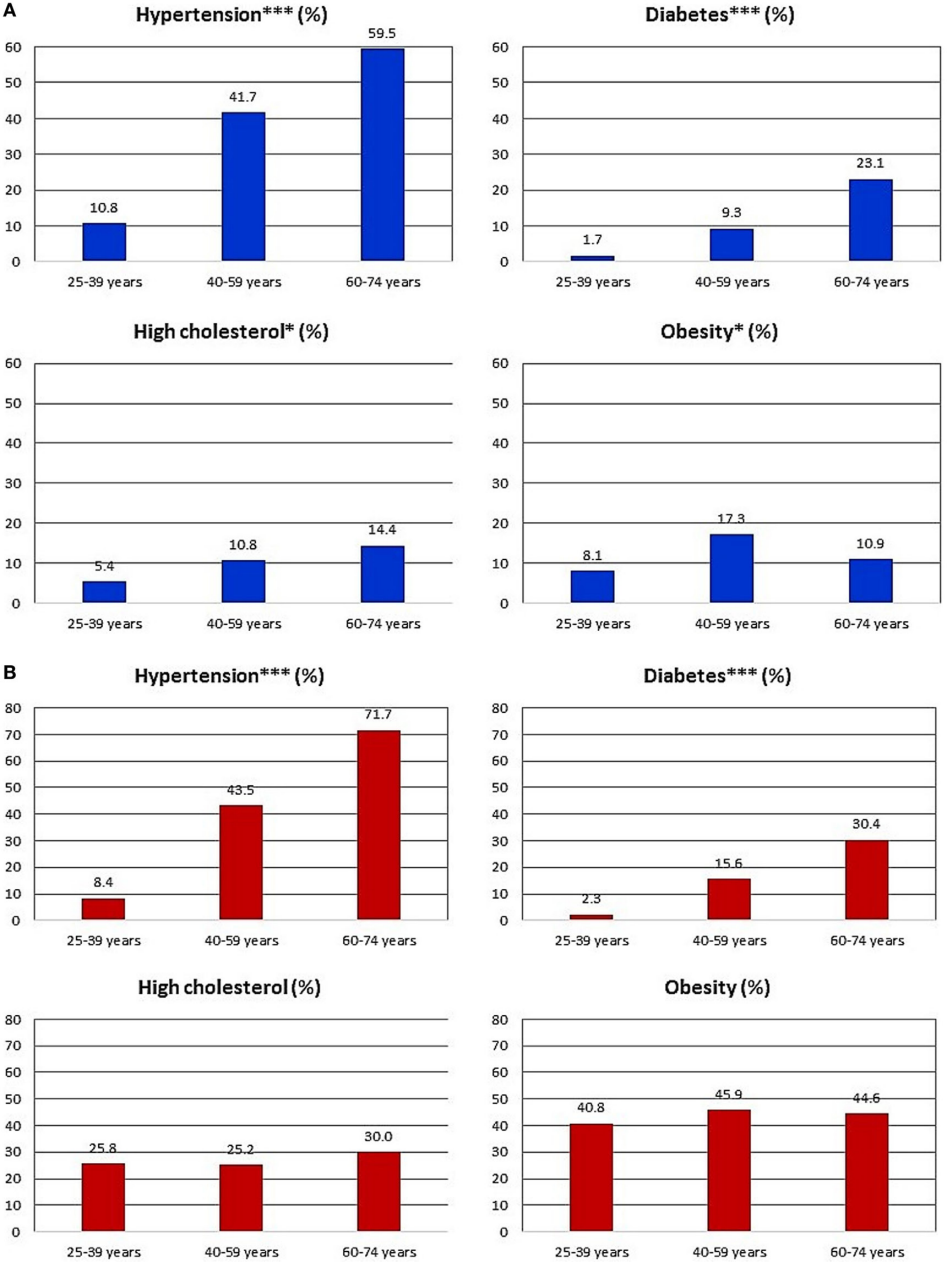
**(A)** Proportion of men with individual cardiovascular disease (CVD) risk factors various within age categories. *p < 0.05; **p < 0.01; ***p < 0.001 for difference in proportion across age categories, derived from chi-squared tests. Proportions are weighted for complex survey design to provide nationally representative (population) estimates. Percentages shown reflect the weighted estimates and not the simple proportion of participants based on observed numbers. *n* = 237 for 25–39 years age group, 285 for 40–59 years age group, and 156 for the 60–74 years age group. **(B)** Proportion of women with individual CVD risk factors various within age categories. *p < 0.05; **p < 0.01; ***p < 0.001 for difference in proportion across age categories, derived from chi-squared tests. Proportions are weighted for complex survey design to provide nationally representative (population) estimates. Percentages shown reflect the weighted estimates and not the simple proportion of participants based on observed numbers. *n* = 600 for 25–39 years age group, 677 for 40–59 years age group, and 276 for the 60–74 years age group.

Sex-specific prevalence estimates for each CVD risk factor within education categories are shown in Figures 1A,B. There were statistically significant associations with education level for all the CVD risk factors except for high cholesterol among men. Prevalence of the CVD risk factors was generally lowest among persons with post-secondary education. Similarly, for most outcomes, prevalence was highest among persons with only primary level education. As expected, CVD risk factor prevalence was generally lowest among the younger participants and highest among the oldest participants Figures 2A,B. Additionally, there was a statistically significant association between educational attainment and age with the older participants being more likely to have lower education (*p* < 0.001 for males and females, data not shown).

Table 3 shows the age-adjusted prevalence estimates, prevalence differences, and prevalence ratios for men. The prevalence of hypertension and diabetes was lowest among those with post-secondary education, but for high cholesterol and obesity, prevalence was lowest in the full secondary and primary or less education categories, respectively. Using post-secondary education as the reference group, there was evidence for a statistically significant higher prevalence of diabetes among the lower education groups with prevalence difference ranging from 6.9 to 7.4 percentage points (*p* < 0.05 for each group). Similarly, the prevalence ratios for diabetes compared to the post-secondary education group ranged from 3.3 to 3.5, but none of these achieved statistical significance. Prevalence differences and prevalence ratios for hypertension also suggested higher prevalence for the lower education groups but were not statistically significant. On the other hand, prevalence differences and prevalence ratios for obesity and hypercholesterolemia suggested that prevalence was lower among the less educated groups.

**Table 3:** **Age-adjusted prevalence estimates, prevalence difference, and prevalence ratios for CVD risk factors within education categories for men in the Jamaica Health and Lifestyle Survey 2007–2008**.

Characteristic	Post-secondary *N* = 64	Full secondary *N* = 268	Junior secondary *N* = 200	Primary or less *N* = 146
Prevalence	% (95% CI)	% (95% CI)	% (95% CI)	% (95% CI)

Hypertension	28.4 (15.9–41.0)	33.0 (26.9, 39.1)	35.0 (27.7, 42.3)	37.3 (29.8, 44.9)
Diabetes	3.0 (0, 7.1)	10.2 (5.3, 15.1)	9.8 (5.5, 14.1)	10.4 (6.4, 14.4)
High cholesterol	12.1 (0, 24.5)	8.8 (4.5, 13.2)	10.6 (5.6, 15.7)	8.9 (4.0, 15.8)
Obesity	14.0 (3.2, 24.8)	24.2 (16.3, 32.2)	8.7 (4.9, 12.5)	6.3 (2.8, 9.7)

Prevalence difference		% (95% CI)	% (95% CI)	% (95% CI)

Hypertension	Reference	4.6 (−9.3, 18.9)	6.6 (−8.6, 21.8)	8.9 (−6.2, 24.0)
Diabetes	Reference	7.2 (1.1, 13.3)*	6.9 (0.9, 12.8)*	7.4 (1.4, 13.5)*
High cholesterol	Reference	−3.3 (−16.6, 9.9)	−1.5 (−15.0, 12.0)	−3.2 (−18.7, 12.3)
Obesity	Reference	10.2 (−3.9, 24.3)	−5.3 (−16.5, 5.8)	−7.8 (−18.9, 3.3)

Prevalence ratio		PR (95% CI)	PR (95% CI)	PR (95% CI)

Hypertension	Reference	1.16 (0.72, 1.86)	1.23 (0.74, 2.05)	1.31 (0.80, 2.16)
Diabetes	Reference	3.43 (0.82, 14.4)	3.31 (0.76, 14.4)	3.51 (0.79, 15.5)
High cholesterol	Reference	0.73 (0.23, 2.29)	0.88 (0.28, 2.73)	0.74 (0.18, 3.02)
Obesity	Reference	1.73 (0.73, 4.11)	0.62 (0.27, 1.45)	0.45 (0.18, 1.11)

*Estimates were derived from Poisson regression models. All models adjusted for age as categorical variable. Separate models were created for each CVD risk factor. Age-adjusted prevalence estimates and prevalence difference were derived from the models using post-estimation commands. Number of participants and confounders included for each model were as follows: hypertension—631 participants; adjusted for BMI, glucose, and cholesterol; diabetes—678 participants; adjusted for BMI and SBP; high cholesterol—636 participants; adjusted for BMI, glucose, and SBP; obesity—678 participants; adjusted for cholesterol and SBP*.

*p < 0.05; for difference in proportion compared to reference category (post-secondary education) derived from post-estimation tests.

*BMI, body mass index; SBP, systolic blood pressure; CVD, cardiovascular disease*.

Table 4 shows the age group-specific prevalence estimates, prevalence differences, and prevalence ratios for CVD risk factors for women. Among the younger women (25–39 years old age group), prevalence of CVD risk factors was generally lowest among those with post-secondary education, with statistically significant prevalence differences for hypertension, hypercholesterolemia, and obesity. A similar pattern was seen with the prevalence ratios. The magnitude of the prevalence ratios suggested large disparities, with prevalence ratios ranging between 2.5, comparing persons with full secondary level education to those with post-secondary education for hypertension, and 26.5, comparing those with only primary level education to those with post-secondary education for diabetes. The confidence intervals were relatively wide given the smaller number of participants in these subgroups. Among middle-aged (40–59 years) and older women (60–74 years), the patterns were less consistent. For high cholesterol and obesity, prevalence of CVD risk factor was often highest among those with higher education level (post-secondary or full secondary). For hypertension, prevalence was significantly higher among those with less education for women in the 60–74 age group but not in the middle-aged women. For diabetes mellitus, prevalence was generally higher among the less educated but did not achieve statistical significance.

**Table 4:** **Age-specific prevalence estimates, prevalence difference, and prevalence ratios for CVD risk factors within education categories for women in the Jamaica Health and Lifestyle Survey 2007–2008**.

Characteristic	Post-secondary *N* = 136	Full secondary *N* = 734	Junior secondary *N* = 411	Primary or less *N* = 272
Prevalence	% (95% CI)	% (95% CI)	% (95% CI)	% (95% CI)

**Hypertension**				
25–39 years	4.0 (1.4, 6.6)	10.0 (7.1, 12.9)	16.9 (9.2, 24.6)	15.7 (0, 43.4)^a^
40–59 years	38.4 (24.6, 52.2)	36.1 (29.6, 42.7)	46.3 (38.7, 53.8)	55.0 (43.6, 66.9)
60–74 years	42.2 (30.1, 54.3)	87.8 (72.3, 100)	65.7 (53.3, 78.2)	72.7 (64.9, 80.6)

**Diabetes**				
25–39 years	0.8 (0, 2.2)	2.7 (1.1, 4.3)	4.6 (0.3, 8.9)	20.1 (0, 45.1)
40–59 years	8.6 (0, 17.3)	14.3 (10.6, 18.1)	16.0 (11.1, 21.0)	21.7 (11.4, 31.9)
60–74 years	18.5 (0, 38.6)	36.0 (13.1, 59.0)	27.2 (16.0, 38.5)	30.5 (22.4, 38.6)

**High cholesterol**				
25–39 years	1.6 (0.3, 3.0)	36.9 (34.6, 39.1)	13.1 (4.3, 22.0)	23.7 (0, 55.5)
40–59 years	37.0 (21.2, 52.8)	23.2 (18.7, 27.6)	25.1 (19.0, 31.2)	26.2 (15.6, 36.5)
60–74 years	23.5 (0, 48.8)	26.4 (11.0, 41.8)	28.4 (16.4, 40.4)	29.9 (22.4, 37.5)

**Obesity**				
25–39 years	7.8 (4.2, 11.5)	34.0 (29.3, 38.7)	49.4 (34.6, 64.2)	37.2 (4.7, 69.6)
40–59 years	60.3 (43.3, 77.4)	46.3 (40.6, 52.0)	41.4 (34.3, 48.7)	42.4 (32.8, 51.9)
60–74 years	53.5 (29.0, 78.0)	27.2 (13.7, 40.8)	38.1 (27.8, 48.4)	42.2 (31.8, 52.6)

Prevalence difference		% (95% CI)	% (95% CI)	% (95% CI)

**Hypertension**				
25–39 years	Reference	6.0 (2.2, 9.9)**	12.9 (4.8, 21.0)***	11.7 (−1.6, 39.6)
40–59 years	Reference	−2.2 (−17.6, 13.1)	7.9 (−8.2, 23.9)	16.6 (−1.6, 34.7)
60–74 years	Reference	45.6 (25.0, 66.1)***	23.6 (4.6, 42.6)*	30.5 (−15.4, 45.7)***

**Diabetes**				
25–39 years	Reference	2.0 (−0.3, 4.2)	3.9 (−0.7, 8.4)	19.3 (−5.7, 44.3)
40–59 years	Reference	5.7 (−3.8, 15.3)	7.5 (−2.5, 17.5)	13.1 (−0.2, 26.4)
60–74 years	Reference	17.5 (−13.9, 48.2)	8.7 (−14.6, 32.0)	12.0 (−10.8, 34.8)

**High cholesterol**				
25–39 years	Reference	35.3 (32.9, 37.7)***	11.5 (2.6, 20.5)*	22.1 (−9.6, 53.9)
40–59 years	Reference	−13.8 (−30.7, 3.0)	−11.9 (−28.7, 4.9)	−10.8 (−30.0, 8.4)
60–74 years	Reference	2.9 (−26.5, 32.3)	4.9 (−24.0, 33.8)	6.4 (−20.7, 33.5)

**Obesity**				
25–39 years	Reference	26.7 (20.3, 32.0)***	41.5 (25.9, 57.2)***	29.3 (−3.7, 62.3)
40–59 years	Reference	−14.0 (−31.1, 3.1)	−18.8 (−37.3, −0.3)*	−17.9 (−37.2, 1.4)
60–74 years	Reference	−26.2 (−53.7, 1.3)	−15.4 (−41.3, 10.5)	−11.2 (−37.1, 14.5)

Prevalence ratio		PR (95% CI)	PR (95% CI)	PR (95% CI)

**Hypertension**				
25–39 years	Reference	2.50 (1.25, 5.03)**	4.23 (1.96, 9.15)***	3.93 (0.60, 25.7)
40–59 years	Reference	0.94 (0.63, 1.40)	1.20 (0.81, 1.79)	1.43 (0.95, 2.16)
60–74 years	Reference	2.08 (1.47, 2.94)***	1.56 (1.08, 2.25)*	1.72 (1.26, 2.36)***

**Diabetes**				
25–39 years	Reference	3.58 (0.45, 28.3)	6.10 (0.71, 52.7)	26.5 (2.90, 242.4)**
40–59 years	Reference	1.67 (0.59, 4.76)	1.87 (0.65, 5.37)	2.53 (0.84, 7.58)
60–74 years	Reference	1.95 (0.56, 6.79)	1.47 (0.46, 4.68)	1.65 (0.53, 5.16)

**High cholesterol**				
25–39 years	Reference	22.7 (10.1, 51.1)***	8.1 (2.82, 23.2)***	14.6 (3.17, 67.4)***
40–59 years	Reference	0.63 (0.39, 1.01)	0.68 (0.42, 1.10)	0.71 (0.39, 1.27)
60–74 years	Reference	1.12 (0.34, 3.72)	1.21 (0.37, 3.90)	1.27 (0.42, 3.88)

**Obesity**				
25–39 years	Reference	4.34 (2.69, 6.99)***	6.30 (3.55, 11.2)***	4.74 (1.72, 13.1)**
40–59 years	Reference	0.77 (0.58, 1.02)	0.69 (0.50, 0.95)*	0.70 (0.49, 0.998)*
60–74 years	Reference	0.51 (0.27, 0.98)*	0.71 (0.43, 1.18)	0.79 (0.48, 1.29)

*Estimates were derived from sex-specific Poisson regression models which included an interaction term for the age group and education category interaction. Separate models were created for each CVD risk factor. Age-specific prevalence estimates and prevalence difference were derived from the models using post-estimation commands. Number of participants and confounders included for each model were as follows: hypertension—1,442 participants; adjustment for BMI, glucose, and cholesterol; diabetes—1,451 participants, adjusted for BMI and SBP; high cholesterol—1,443 participants, adjusted for BMI, glucose, and SBP; obesity—1,451 participants, adjusted for cholesterol and SBP*.

*^a^Lower confidence limits for prevalence estimates were reported as zero (0) if calculated values were negative*.

*p < 0.05; **p < 0.01; ***p < 0.001 for prevalence difference ≠ 0 or prevalence ratio ≠ 1 when compared to reference category (post-secondary education) derived from post-estimation tests.

*BMI, body mass index; SBP, systolic blood pressure; CVD, cardiovascular disease*.

*Number of participants in each age subgroup for post-secondary, full secondary, junior secondary, and primary or less were as follows: 25–39 years: 77, 399, 113, 11; 40–59 years: 46, 317, 216, 98; 60–74 years: 13, 18, 82, 163*.

## Discussion

In this study, we have shown that patterns of educational health disparity varied by sex and by age group among women. Statistically significant disparities were seen for diabetes mellitus among men and for all the CVD risk factors studied among younger (25–39 years old) women. Among men, persons in the lower education categories had higher prevalence of hypertension and diabetes, while less educated young women had higher prevalence of all four CVD risk factors. The point estimates suggested that men with higher educational attainment may have higher prevalence of obesity and hypercholesterolemia, while among older women, those with higher educational attainment may have higher prevalence of obesity, but these associations were not statistically significant.

The findings of this study show some differences when compared to previous studies from Jamaica and some other developing countries (19–22, 24, 25). In fact, the patterns of disparity are now beginning to resemble that seen in more developed countries. For example, Ferguson and colleagues (25) found that the prevalence of the metabolic syndrome was highest among men with post-secondary education, but among women, the prevalence was highest among those with primary or less education. Similarly, Mendez and colleagues (22) found that men with higher income were more likely to be obese, while obesity levels were high among women, even among those with very low income. While the sex differences in the pattern of disparity has remained, we now report significant differences by age group among women and a pattern of disparity for diabetes and hypertension among men now more consistent with that seen in developed countries, with higher prevalence in the lower education groups. The pattern of disparity for obesity and hypercholesterolemia among men remain similar to the earlier studies with higher prevalence among those with higher educational attainment, but for women, this is now seen only for obesity among the older age cohorts. Our findings are somewhat similar to those reported by Jones-Smith and colleagues in a study from China (41). In their study, Jones-Smith and colleagues reported that while there were no significant educational health disparities among Chinese men and women in 1989, in 2006, significant disparities had emerged with women with higher education having less overweight/obesity while men with higher education having more overweight/obesity (41).

When we compared our findings to those reported from developed counties, we found that there were some similarities. In the United States, lower education is generally associated with higher prevalence of CVD risk factors in both men and women (2, 42). Recent data from the Centers for Disease Control and Prevention show that diabetes prevalence was lowest among persons in the highest education categories in both 2006 and 2010, but the report did not discuss sex- or race-specific educational disparities (43). With regards to obesity, there were inverse associations with education in both men and women, with the lowest prevalence among those who were college graduates (44).

The differences seen in patterns of disparity by sex between countries such as the United States and developing countries such as Jamaica and China may be a result of differences in the stage of the epidemiological transition and how these factors influence the social shaping of population health, where CVD has been shown to be associated with affluence in the early stages of the epidemiological transition but with lower SES later on (4, 19). The fact that age group differences were seen among women, with the younger women showing disparity patterns more consistent with developed countries also support the notion that this reflects ongoing epidemiological transition. Other factors such as health literacy, health seeking behavior, physical activity levels, and social stigma associated with obesity in women may be other contributory factors (41).

This study had a number of strengths. First, data were from a nationally representative sample and used weighting procedures to adjust for differences between sample distribution and population distribution; the findings can therefore be generalized to the Jamaican population and would have implications for similar developing countries especially in the Caribbean. We also report category-specific prevalence estimates, prevalence ratios, and prevalence differences thus allowing for detailed analysis of the disparity patterns. Additionally, we report on four CVD risk factors thus facilitating comparison of similarities and differences between risk factors and whether the education effect showed heterogeneity across risk factors.

Study limitations include the fact that only one time point was evaluated thus precluding analysis of temporal trends in disparity patterns. Additionally, relatively small numbers in some educational categories may have resulted in insufficient power to show statistical significance for some of the differences seen. Small numbers in some of the age and sex subgroups also resulted in relatively imprecise estimates with wide confidence intervals. We also acknowledge that there were missing data for some participants which could have influenced the findings. However, the proportion with missing data was relatively small (≈4%) and except for a lower prevalence of hypertension there were no significant differences in the prevalence of the CVD risk factors evaluated or in the distribution of education categories. It is therefore unlikely that the exclusion due to missing data would have had a significant impact on our results.

## Conclusion

In Jamaica, there are disparities in the prevalence of CVD risk factors, with different patterns among men and women, and by age group among women. Higher education is associated with lower prevalence of diabetes among men and with lower prevalence of all four CVD risk factors among younger women. Patterns of disparity are somewhat different from that seen in earlier studies from Jamaica, possibly due to ongoing epidemiological transition. Further research should monitor disparity trends and seek to better understand the reasons for the patterns seen and identify opportunities for intervention.

## Ethics Statement

The study protocol was reviewed and approved by the Ethics Committees of the Faculty of Medical Sciences of the University of the West Indies, Mona, and the Ministry of Health, Jamaica. All participants provided written informed consent.

## Author Contributions

TF contributed to conception and design of study; contributed to the analysis and interpretation of data; drafted manuscript; and critically revised manuscript for intellectual content. NY-C contributed to conception and design of study; lead statistical analyses, and contributed to interpretation of data; and critically revised manuscript for intellectual content. MT-R, IH, NB, AB, and AH contributed to design of study; contributed to interpretation of data; and critically revised manuscript for intellectual content. DF contributed to data collection; contributed to design of study; contributed to interpretation of data; and critically revised manuscript for intellectual content. SM contributed to data collection; contributed to interpretation of data; and critically revised manuscript for intellectual content. MM contributed to conception and design of study; critically revised manuscript for intellectual content. RW lead parent study; supervised data collection and study execution; contributed to conception and design of study; contributed to interpretation of data; and critically revised manuscript for intellectual content. EH and LS contributed to conception and design of study; critically revised manuscript for intellectual content.

## Disclaimer

The findings and conclusions in this report are those of the authors and do not necessarily represent the official position of the Pan American Health Organization.

## Conflict of Interest Statement

The authors declare that the research was conducted in the absence of any commercial or financial relationships that could be construed as a potential conflict of interest.
